# A retrospective study on adjuvant chemotherapy in retinoblastoma: validation of the new recommendation against treatment for pT2a tumors based on the 8th AJCC classification

**DOI:** 10.1186/s12886-024-03585-5

**Published:** 2024-07-24

**Authors:** Hind Manaa Alkatan, Alaa Almuzaini, Hala A. Helmi, Azza MY Maktabi

**Affiliations:** 1 Department of Ophthalmology, College of Medicine, King Saud University, Riyadh, Saudi Arabia; 2 Department of Pathology, College of Medicine, King Saud University, Riyadh, Saudi Arabia; 3 King Saud University Medical City, King Saud University, Riyadh, Saudi Arabia; 4 Pediatric Ophthalmology Division, King Khaled Eye Specialist Hospital, Riyadh, Saudi Arabia; 5 Department of Ophthalmology, McGill University, Montreal, Canada; 6 Ophthalmology Department, King Faisal Specialist Hospital and Research Center, Riyadh, Saudi Arabia; 7 Pathology and Laboratory Medicine Department, King Khaled Eye Specialist Hospital, Riyadh, Saudi Arabia

**Keywords:** Retinoblastoma, Adjuvant chemotherapy, AJCC classification, Histopathology, pT2a, Enucleation, Outcome, Metastasis

## Abstract

**Background:**

Retinoblastoma (RB) is an intraocular malignant tumor detected in early childhood with variable global impact. Histopathological classification of the tumor in enucleated globes with RB is the key for the decision of adjuvant chemotherapy use. We aim to validate the use of adjuvant chemotherapy in cases with combined pre-laminar/intralaminar optic nerve (ON) invasion and focal choroidal invasion according to the American Joint Committee on Cancer (AJCC) 8th classification.

**Methods:**

This is a retrospective study conducted at King Abdulaziz University Hospital (KAUH) and King Khalid Eye Specialist Hospital (KKESH) in Riyadh, Saudi Arabia of all RB cases who underwent enucleation over 22 years (2000 to 2021). The histopathological findings were reviewed to identify the enucleated globes classified as pT2a tumors, as an inclusion criterion. Basic demographic and clinical data were collected via chart review Simple descriptive and basic statistical analysis of the data was used where applicable.

**Results:**

Thirty-one patients who had an enucleated globe with RB that fit into the above classification were included. Sixteen were males and 15 were females. The median age was 14 months (IQR = 14 months). Most of the patients (93.5%) had no family history of RB. The commonest presentation was leukocoria in 87.1% followed by squint in 32.3%. Fourteen patients (45.2%) were treated by enucleation alone while 17 patients (54.8%) received adjuvant chemotherapy. Out of these, 7 patients had unilateral RB and the remaining 10 patients had bilateral RB. None of our patients developed recurrence or metastatic disease irrespective of the indication for adjuvant chemotherapy use after a maximum period of follow up reaching 17.84 years and a median of 10.6 years (IQR = 5.92).

**Conclusions:**

In patients with 8th AJCC histopathological classification of pT2a, chemotherapy following enucleation might not be justified. The outcome in our untreated group of patients did not differ from the treated group with the absence of metastasis after a relatively long period of follow up with a median exceeding 10 years in both groups. Therefore, the risk and benefit of post enucleation adjuvant chemotherapy in the treatment of unilateral RB should be carefully decided and discussed with the primary caregivers taking into consideration the most recent evidence and recommendations in the literature.

## Introduction

Retinoblastoma (RB) is a malignant intraocular tumor detected in early childhood with variable morbidity and mortality depending on the geographical distribution and available treatment modalities. Its prognosis is highly dependent on the management strategies. While the decision for adjuvant chemotherapy after enucleation in advanced cases is crucial, it has been controversial in tumours with less risk features. As observed in the changes in the recent American Joint Committee on Cancer (AJCC) 8th classification, the criteria for chemotherapy after enucleation might have been changing. In Saudi Arabia, the histopathological risk features of RB enucleated globes have been studied based on the AJCC 8th classification with string recommendation to have this newer classification followed to unify the treatment protocols. [1] The same group also studied the value of chemo-reduction prior to enucleation and found that secondarily enucleated globes had lower tumour classification categorization with statistical significance compared to primarily enucleated globes. [2] In these studies, the common recommendation was in favour of using adjuvant chemotherapy in cases with high histopathological risk features (pT2b or worse according to the 7th edition, which is equivalent to pT2a or worse according to the 8th edition). [1, 3] More recent recommendations from well-known RB centres in North America have come up with suggested modifications of the above older recommendation especially with the implementation of the AJCC 8th classification, where authors considered adjuvant chemotherapy not to be indicated for cases with combined focal choroidal invasion and prelaminar/intralaminar ON invasion. [4] However, no previous study was conducted to confirm this based on the observed outcome between the group that received adjuvant chemotherapy and the one that did not. This study aimed to validate the use of adjuvant chemotherapy in cases with combined ON invasion and focal choroidal invasion.

## Methods

This retrospective chart review study was performed at KAUH and KKESH in Riyadh, Saudi Arabia. Initially, all RB cases who underwent enucleation between the year 2000 to 2021 were collected. The corresponding histopathological slides were reviewed to confirm the histopathological findings and classify the high-risk RB features according to the newly applied AJCC 8th classification. The inclusion criteria was to include any patient with RB who underwent enucleation with available specimen sent to the Histopathology laboratory and the tumor fits into stage pT2a according to the 8th AJCC classification within the study period The Exclusion criteria included RB patients who did not require enucleation, bilateral RB cases with advanced tumor stage (worse than pT2a) in one of the eyes, patients with regional lymph node involvement and/or evidence of intracranial or distant metastasis.

The study received expedited approval by the Research IRB and Human Ethics Committee at KKESH (RP-22,072-R) on 1 June 2020 with collaborative agreement with KAUH and approval by the Research committee in the second institution.

In the first part of the study the gathered demographic data of RB patients was analyzed in addition to the brief analysis of the clinical signs and symptoms. The second part of the study focused on the diagnosis, histopathological description, treatment, classification of RB tumors in the enucleated globes, duration of follow-up, and the outcome.

Data collected were presented using IBM SPSS ver 23 (IBM Corp., Armonk, NY). Categorical and nominal variables were represented through counts and percentages. Continuous variables were represented by mean and standard deviations. To determine relationships between domains, Chi-square test was used assuming normal distribution with a < 0.05 CL to discard the null hypothesis.

## Results

The first part of this study focused on the demographics of the study sample. There was a total of 31 patients during the period of 2000 to 2021 that met the inclusion criteria. Sixteen patients were males and 15 were females. Most of the participants were Saudis (96.8%) while 3.2% were non-Saudi (1 from Yamen and 1 from Syria). The age range at the time of enucleation was 2 to 61 months, with an average age of 16 months and a median of 14 months (Q1 = 8, Q3 = 22, IQR = 14 months). Among these patients, 29 (93.5%) had no family history of retinoblastoma.

Clinically (Table 1), most of the patients presented initially with leukocoria (87.1%) followed by squint (32.3%). Other symptoms included megalo-cornea and red eye in 1 patient each. Clinical signs upon examination showed similar pattern with leukocoria in 87.1%, followed by strabismus, and one patient was found to have buphthalmos. Additionally, neovascular glaucoma and ciliary injection were noted in 1 patient. Poor vision with absent fixation of the affected eye at initial presentation was recorded in most (20/31) of the cases (64.5%), 2 patients were able to fix and follow with the affected eye (6.5%), and one patient (3.2%) had no light perception (NLP). The vision of the affected eye was not documented clearly in the files of the remaining 8/31 patients (25.8%).

**Table 1:** The clinical symptoms and signs in order of frequency in the 31 patients who were included in the study

Variables		Count	%
Total		31	100.0
Symptoms	Leukocoria	27	87.1
	Squint	10	32.3
	Proptosis	0	0.0
	Megalo-cornea	1	3.2
	Ciliary injection	1	3.2
Clinical sign	Leukocoria	27	87.1
	Squint	7	22.6
	Proptosis	0	0.0
	Hyphema	0	0.0
	Buphthalmos	1	3.2
	Neovascular glaucoma	1	3.2
	Ciliary injection	1	3.2

Right eye was involved in 7 patients (22.6%), while 10 patients (32.3%) had left eye involvement, and 14 (45.2%) had bilateral retinoblastoma. Out of the 14 patients with bilateral retinoblastoma, 1 patient only underwent bilateral enucleation, however, the other enucleated globe did not meet the inclusion criteria, thus was not included in the cohort. Clinical classification of the tumors was 3.2% group C, 12.9% group D, and 83.9% group E, at presentation of the affected enucleated eye. In bilateral cases, the classification of the contralateral eye was clinically classified as group A in 3 patients (21%), group B in 6 patients (43%) while 2 patients only were classified in each of the group’s C and D (14%), and 1 patient in group E.

The histopathological characteristics of the 31 enucleated globes are summarized in Table 2.

**Table 2:** Summary of the histopathological characteristics in the 31 enucleated globes

Variables		Count		%
Optic nerve (ON) invasion	Pre-laminar		18	58.1
	Laminar		13	41.9
ON surgical margin	Negative margin		31	100.0
Choroidal Invasion	Focal		31	100.0
Scleral/Extra-scleral invasion	Absent		31	100.0
Retinoblastoma seeding	No seeding		3	9.7
	Vitreous		18	58.1
	Subretinal		24	77.4
	Sub RPE		23	74.2
Tumor differentiation	Undifferentiated		7	22.6
	Poor		8	25.8
	Moderate		12	38.7
	Well		3	9.7
	Difficult to assess or unknown		1	3.2
Tumor size within the posterior cavity	1/3 − 1/2		6	19.4
	> 1/2		12	38.7
	Whole		13	41.9
Growth pattern	Endophytic		11	35.5
	Exophytic		16	51.6
	Combined		4	12.9
Origin from the retina	Inner layer		6	19.4
	Outer layer		7	22.6
	Both		4	12.9
	Unknown		14	45.2
Calcification	Minimal		10	32.3
	Prominent		19	61.3
	None		2	6.5
Necrosis	< 20%		1	3.2
	20–40%		11	35.5
	40–60%		11	35.5
	> 60%		8	25.8
Mitotic figures	Rare		3	9.7
	Occasional		3	9.7
	Frequent		17	54.8
	Numerous		8	25.8
Focality	Unifocal		12	38.7
	Multifocal		19	61.3

**Fig. 1:**
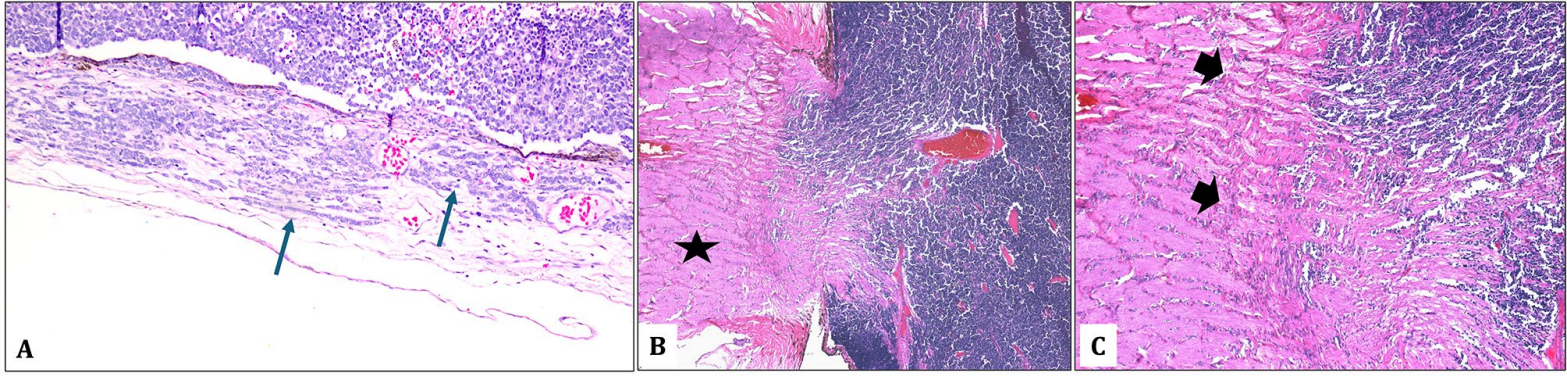
**A**: An enucleated globe with true choroidal invasion (black arrows) where the tumor cells are seen within the choroid and should measure less than 3 mm (Original magnification x400 Hematoxylin and eosin). **B**: Another enucleated globe with optic nerve (black star) showing undifferentiated retinoblastoma and prelaminar optic nerve invasion (Original magnification x100 Hematoxylin and eosin). **C**: Higher magnification of the same globe showing the level of optic nerve invasion which does not reach the lamina cribrosa (black arrow heads) thus considered to be prelaminar (Original magnification x400 Hematoxylin and eosin)

All patients underwent enucleation and were all classified histopathologically according to 8th AJCC as pT2a with demonstration of focal choroidal invasion and prelaminar optic nerve invasion in Fig. 1. Out of these, 17 patients (54.8%) received adjuvant chemotherapy after enucleation. Of these 17 patients who received combination therapy 7 patients had unilateral RB and 10 patients received adjuvant chemotherapy as part of their therapy for bilateral RB. The remaining 45.2% who did not receive adjuvant chemotherapy, were mostly old cases that were diagnosed and managed before the implementation of 7th AJCC classification (38.7%) while parents of one patient (3.2%) declined the chemotherapy and in another patient, chemotherapy was not given based on the new recommendation in more recent literature. This is summarized in Table 3.

**Table 3:** Summary of the 2 groups of patients, the ones who received adjuvant chemotherapy after enucleation and the other group who did not

Variable		Number	Percentage
Adjuvant systemic chemotherapy after enucleation	Yes	17	54.8%
	No	14	45.2%
The reason for not receiving adjuvant chemotherapy	Before implementing 7th AJCC classification	12	38.7%
	Refusal by guardians	1	3.2%
	New recommendation in the literature	1	3.2%

The follow up period ranged from 1.13 to 17.84 years with a mean of 9.66 ± 4.2 years and a median of 10.6 years (Q1 = 6.25. Q3 = 12.17, IQR = 5.92 years). None of the patients in the group who received adjuvant chemotherapy following enucleation (17/31) and the group who did not (14/31) developed recurrence or metastatic disease. In the first group, 10 patients had bilateral RB and received adjuvant chemotherapy as continuation of their therapy, out of which 9 regressed and one ended with enucleation of the other eye. When histopathological variables were compared between the 2 groups, subretinal seeding was the only finding that was correlated to the group that did not receive adjuvant therapy with a statistically significant *p* value of *0.031* (significant using Chi-Square at < 0.05 level). When the same variables were correlated with the clinical findings, presence of calcification was the only variable that had significant correlation with squint as a presenting symptom *p* = 0.025 (using Chi-Square at < 0.05 level).

## Discussion

The outcome and prognosis in RB have improved with the evolving variety of therapeutic options that mainly depends on the use of chemotherapy either for the purpose of chemo-reduction prior to enucleation or as an adjuvant therapy for histopathologically defined high-risk tumors. ^{2}^ Adjuvant treatment protocols has been variable from one center to another, and newer studies have been more recently published to better identify and classify the risk factors in enucleated globes for better development of treatment strategies that weighs the risks versus the benefits of used chemotherapy. In unilateral advanced retinoblastoma, primary enucleation is an efficient therapy. Aerts in a prospective study of the overall survival in unilateral retinoblastoma treated by primary enucleation, demonstrated excellent outcome even in the group with minimal or no choroidal invasion and/or prelaminar or no optic nerve invasion. This group of patients in their study did not receive adjuvant chemotherapy. However, it has been debatable if focal choroidal invasion only when combined with pre-laminar and/or intralaminar infiltration of the ON would be considered as low-risk features and would not necessitate the use of post-enucleation adjuvant chemotherapy [4–6]. The role of post-enucleation adjuvant therapy in unilateral RB patients is to inhibit both local and distant relapses and is achieved, but yet it is believed that the management of retinoblastoma should be carefully designed depending on regional variability of treatment methods and patient’s characteristics including germline mutation and histopathological risk factors [6, 7]. 

In our center, the 6th followed by the 7th AJCC classifications was implemented in the years 2009 then 2010 respectively. The enucleated globes with combined focal choroidal invasion and ON invasion (even if prelaminar or intralaminar) were classified as high-risk features (pT2b) as an indication for adjuvant chemotherapy according to our more cautious protocols during that period. This has changed with the evolved 8th classification and the justification for this post-enucleation treatment was re-visited with the commencement of its implementation in our centers in the year 2019 since the RB tumor with this type of ON invasion was considered to be still intraocular in nature with low risk for metastatic disease (classified as pT2a). The oncology group study in the same year have indicated that this level of ON involvement even when associated with focal choroidal invasion may not justify the use of adjuvant chemotherapy. The European Retinoblastoma group (EURbG) have also reported in 2020 inconsistency in the protocol for adjuvant chemotherapy in RB treatment since 84.4% of their centers have defined focal choroidal invasion or prelaminar ON invasion as low risk features while 8/26 centers considered the 2 combined types of invasions above as a justification for treatment with adjuvant chemotherapy (in a similar way to our initially followed guidelines during the era of the 7^Th^ AJCC implementation) [8] We had the chance of studying our cases that have fallen into this classification in 31 eyes to validate their recommendation. The demographics in this selected group were similar to a larger study on enucleated globes in advanced RB in the same geographical area with a mean age of 16.74 months at enucleation, equal gender distribution, and negative family history in 93.5% of the cases [1]. 

The clinical manifestations were also comparable to other studies, with leukocoria being the commonest in 87.1% followed by squint, which was found to be significantly correlated to the presence of calcification. Histopathologically all enucleated globes had either prelaminar or intralaminar ON invasion in 58.1% and 41.9 respectively as well as focal choroidal invasion only as per our defined inclusion criteria. Alkatan et al., have concluded that less tumor differentiation constituted a relative risk for massive choroidal invasion and in our group of cases, the tumor was mostly moderately differentiated in about 40%, which may correlate with the fact that all of our globes had only focal choroidal invasion [1]. In contrary to the same study, multifocal tumors were found in less number of globes in this particular cohort (61.3% in our study compared to 83%). The focus of our study was to identify the group of patients with unilateral RB who did not receive adjuvant chemotherapy (Table 3) either because they were diagnosed and treated before the implementation of our treatment protocol (in 12/14), or refusal (in 1/14) or based on the more recent literature against such protocol (in 1/14). Out of the remaining 17 cases who received adjuvant chemotherapy, 10 patients were bilateral and had to continue their chemotherapy. When we compared the outcome of both groups, none of the patients had local tumor recurrence nor metastasis irrespective of whether they received adjuvant chemotherapy or not after an average follow up period of 9.66 ± 4.2 years at the time of the data collection and analysis of this study. Metastasis from retinoblastoma normally happens within one year of retinoblastoma detection. If there is no metastatic disorder by five years after retinoblastoma treatment, the patient is typically judged to be cured. [9–11] The EURbG study on adjuvant therapy mentioned the benefit of RB biomarkers in correlation with metastatic relapse in high-risk RB patients and recommended the consideration of adding these biomarkers and possibly radiological indicators as well to the known histopathological risk factors to come up with a unified treatment guidelines across Europe. [8] Even though it was not our sole choice to treat or not but the outcome in these 14 patients would lead us to the conclusion that the 7 cases of unilateral RB in the other group might have been over treated. The small number of patients that were included might be considered as a limitation in our study, however, the absence of metastatic disease in our patients after a relatively long period of follow up, which exceeds 5 years in average is still significant and these patients are considered to be cured. This is expected to constitute a limitation in any future prospective studies on this topic considering the less number of RB patients undergoing primary enucleation since the successful use of intraarterial and intravitreal chemotherapy. [8] In all cases, detailed risk-benefit analysis must be discussed with the primary caregivers and optimizing patients health must be prioritized [12]. 

## Conclusion

The decision and criteria for the use of adjuvant chemotherapy following enucleation in RB is still controversial and even though it is meant to be indicated for cases with high-risk histopathological features, the treatment plan must be well-adjusted with explanation of possible adverse effects such as transient bone marrow suppression to the parents. Our findings support the previous recommendations of the oncology group study against the use of adjuvant chemotherapy following the enucleation of RB globes with such low-risk features. The absence of recurrence or metastasis observed in our non-treated group after such a long period of follow up, is a strong indication that enucleation alone is effective in unilateral cases that are histopathologically classified as pT2a. Strong universal protocols should be created reflecting these recommendations to be implemented globally.

## Data Availability

The datasets used and analysed during the current study are available from the corresponding author on reasonable request.

## Abbreviations

RB: Retinoblastoma
AJCC: American Joint Committee on Cancer
ON: Optic nerve
KKESH: King Khaled Eye Specialist Hospital
KAUH: King Abdulaziz University Hospital
EURbG: European Retinoblastoma group

## References

[1] Alkatan HM, AlQahtani FS, Maktabi AMY. Enucleated globes with Advanced Retinoblastoma: correlation of histopathological features and reclassification of tumors according to the 8th Edition. (AJCC) Int Ophthalmol 19 March. 2020;40(7):1739–47. 10.1007/s10792-020-01342-3. of the American Joint Commission on Cancer. 10.1007/s10792-020-01342-3 32193778

[2] Alkatan HM, Al-Dahmash SA, Almesfer SA, AlQahtani FS, Maktabi AMY. High-risk features in primary versus secondary enucleated globes with advanced retinoblastoma: a retrospective histopathological study. Int Ophthalmol. 2020;40(11):2875–87. 10.1007/s10792-020-01472-8. Epub 2020 Jul 6. PMID: 32632618. 32632618 10.1007/s10792-020-01472-8

[3] Alkofide A, Alkatan HM, Khafaja Y, Siddiqui K, Jafri R, Ayas M, AlMesfer SA. Post-enucleation retinoblastoma: outcome analysis and evaluation of Prognostic features. J Nat Sci Medicine: J Nat Sci Med January. 2021;4(1):40–5. 10.4103/JNSM.JNSM_58_20. 10.4103/JNSM.JNSM_58_20

[4] Chévez-Barrios P, Eagle RC Jr, Krailo M, Piao J, Albert DM, Gao Y, Vemuganti G, Ali MJ, Khetan V, Honavar SG, O’Brien J, Leahey AM, Matthay K, Meadows A, Chintagumpala M. Study of unilateral Retinoblastoma with and without histopathologic high-risk features and the role of Adjuvant Chemotherapy: A Children’s Oncology Group Study. J Clin Oncol. 2019;37(31):2883–91. Epub 2019 Sep 20. PMID: 31539297; PMCID: PMC6823888. 31539297 10.1200/JCO.18.01808 PMC6823888

[5] Honavar SG, Singh AD, Shields CL, Meadows AT, Demirci H, Cater J, Shields JA. Postenucleation adjuvant therapy in high-risk retinoblastoma. Arch Ophthalmol. 2002;120(7):923 – 31. 10.1001/archopht.120.7.923. PMID: 12096963. 10.1001/archopht.120.7.923 12096963

[6] Aerts I, Sastre-Garau X, Savignoni A, Lumbroso-Le Rouic L, Thebaud-Leculée E, Frappaz D, et al. Results of a multicenter prospective study on the postoperative treatment of unilateral retinoblastoma after primary enucleation. J Clin Oncol. 2013;31(11):1458–63. 23460706 10.1200/JCO.2012.42.3962

[7] Ancona-Lezama D, Dalvin LA, Shields CL. Modern treatment of retinoblastoma: a 2020 review. Indian J Ophthalmol. 2020;68(11):2356–65. 10.4103/ijo.IJO_721_20. PMID: 33120616; PMCID: PMC7774148. 33120616 10.4103/ijo.IJO_721_20 PMC7774148

[8] Pediatric Blood and Cancer. 2021; 68(6): [e28963]. https://doi.org/10.1002/pbc.28963. 10.1002/pbc.28963 33720495

[9] Broaddus E, Topham A, Singh AD. Incidence of retinoblastoma in the USA: 1975–2004. Br J Ophthalmol. 2009;93(1):21–3. 18621794 10.1136/bjo.2008.138750

[10] Kaliki S, Shields CL, Rojanaporn D, Al-Dahmash S, McLaughlin JP, Shields JA, et al. High-risk retinoblastoma based on international classification of retinoblastoma: analysis of 519 enucleated eyes. Ophthalmology. 2013;120(5):997–1003. 23399379 10.1016/j.ophtha.2012.10.044

[11] Kopelman JE, McLean IW, Rosenberg SH. Multivariate analysis of risk factors for metastasis in retinoblastoma treated by enucleation. Ophthalmology. 1987;94(4):371–7. 3587919 10.1016/S0161-6420(87)33436-0

[12] Berry JL, Kogachi K, Murphree AL, Jubran R, Kim JW. A review of recurrent retinoblastoma: children’s Hospital Los Angeles classification and treatment guidelines. Int Ophthalmol Clin. 2019;59(2):65–75. 30908280 10.1097/IIO.0000000000000269 PMC6676476

